# Climate‐Driven Food Loss: The Case of Postharvest Tomato Losses in Southern Tanzania

**DOI:** 10.1002/pei3.70161

**Published:** 2026-06-10

**Authors:** Evodius W. Rutta

**Affiliations:** ^1^ School of Environmental Studies Queen's University Kingston Ontario Canada

**Keywords:** climate change, food loss, postharvest, postharvest management, Tanzania, tomato

## Abstract

Increased weather challenges propelled by climate change are projected to significantly impact postharvest operations, threatening the livelihoods of most small‐scale farmers in several parts of Africa. While research on the effects of climate variability on food production exists, less attention has been given to the impacts of climate change on postharvest management of vegetable crops, especially tomatoes widely produced and consumed on the continent. This study examined the effects of climate variability on the postharvest losses of tomatoes and its implications on the livelihoods of small‐scale tomato farmers in the Kilolo district, southeast Tanzania. Using semi‐structured interviews, focus group discussions, and field observations, data were obtained from 52 (*n* = 52) tomato farmers. Results indicate that unpredictable rains, poor storage conditions, transportation delays, and weather‐related pick‐up delays were major causes of postharvest tomato losses, with farmers reporting losses of up to 40% linked to a lack of storage facilities. Findings also reveal that preharvest tomato losses linked to pest outbreaks were common but not significant compared to postharvest losses caused by poor storage conditions. The study calls for investment in both postharvest crop management training and extension services, and the deployment of low‐cost postharvest infrastructure, especially cold storage and tomato processing facilities, to reduce losses of unsold tomatoes amid weather challenges.

## 1 Introduction

In several developing countries, tomato farming is an important source of livelihood for many rural small‐scale farmers (Arah et al. 2015; FAOSTAT 2014). The global popularity of tomato farming in Africa is attributed to their high‐yielding returns hence attractive to smallholders with limited resources (Arah et al. 2015; Joosten et al. 2015). For consumers, eating fresh tomatoes can significantly boost human health due to their richness in vitamins and antioxidants (Collins et al. 2022; Burton‐Freeman and Reimers 2011). At the global level, China and India are the largest tomato producers, accounting for more than half of global production (FAOSTAT 2014). Other leading producers of tomatoes include the United States of America (USA), Turkey, Egypt, Nigeria, Tunisia, and Morocco. In Africa, besides Egypt and Nigeria, other top tomato‐producing countries include Cameroon, South Africa, Ghana, Kenya, and Tanzania, each with an estimated annual production of over 500,000 t (Arah et al. 2015; MOA and FAO 2018; Obayelu et al. 2014).

In Tanzania, the fresh tomato sector is dominated by small‐scale producers who operate under rain‐fed systems on relatively small parcels of land that are mostly less than 3 ha (MOA and FAO 2018). Annually, Tanzania produces about 600,000 t of fresh tomatoes (MOA and FAO 2018). A large amount of fresh tomatoes is grown in the northern and southern regions of Morogoro, Iringa, Arusha, Mbeya, and the island of Zanzibar (MOA and FAO 2018; Mwagike and Mdoe 2015). Much of the fresh tomatoes are consumed locally, with others exported to Kenya and other neighboring countries in East Africa (MOA and FAO 2018; World Bank 2018). Despite the sector's massive economic potential, tomato producers in Tanzania face several challenges that limit their productivity and growth. Most lack efficient production technologies, rely on rainfed farming, and experience high rates of postharvest losses, leading to low tomato yields (Mashindano et al. 2013; Match Maker 2017; MOA and FAO 2018).

Weather challenges driven by climate variability have also severely disrupted postharvest operations in several tomato‐producing regions in Tanzania (Mwagike and Mdoe 2015; Rutta 2022). Since most farmers lack proper storage facilities, harvested tomatoes spoil even before buyers come for collection due to exposure to heat and sometimes unpredictable rains, often happening during harvest seasons (MOA and FAO 2018; Rutta 2022). To navigate these postharvest challenges, tomato farmers would cover and keep their harvested tomatoes in shaded areas while waiting for buyers. However, these traditional handling and storage techniques can only protect harvested tomatoes for a few hours, resulting in the wastage of unsold tomatoes (Arah et al. 2015; Match Maker 2017). Available studies show that if unresolved, many of these climate‐induced postharvest challenges pose a greater risk to the thriving fresh tomato sector in Tanzania, which has emerged as one of the lucrative agribusiness ventures for many, especially rural youths (Mashindano et al. 2013; Mrema et al. 2008; Rutta 2022).

While the effects of climate change on postharvest management for maize, rice, and other cereals have been widely studied in Tanzania (Abbass et al. 2014; Chegere 2018; Gambwene et al. 2022; Kangalawe et al. 2016; Ndiritu and Ruhinduka 2019), much less attention and research exist on climate change effects on postharvest and supply‐chain challenges for tomatoes and other vegetable crops. To address this gap, this paper examined how climate variability contributes to postharvest tomato losses and its overall impact on farmers' livelihoods in Tanzania.

## 2 Methods

### 2.1 Study Area

**FIGURE 1:**
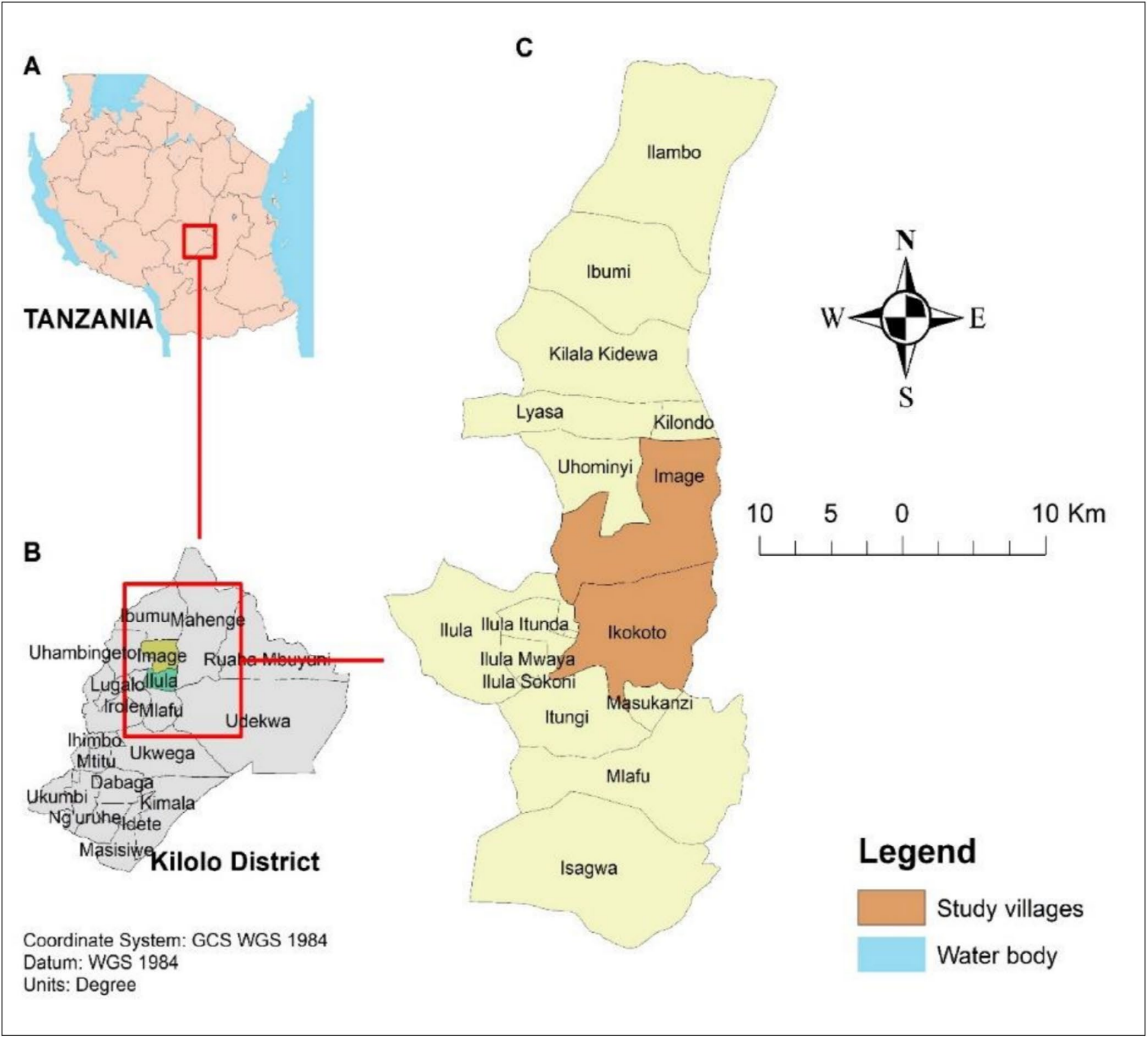
Map of study sites (Rutta 2022).

This study was done in the Kilolo district in the Iringa region of southern Tanzania. Data collection and field visits to tomato farmers were conducted in Image and Ikokoto villages (Figure 1), two of the six villages of Ilula ward. Ilula Ward is an administrative township of the Kilolo district with an estimated population of 26,415 (URT 2016). In 2006, following the booming fresh tomato sector, Ilula Ward was officially pronounced a town by the government of Tanzania under Tanzania's Local Government Act No. 8 of 1982 (Lazaro and Thomsen 2013). Though tomato farming is vital for Ilula's economy, poor postharvest infrastructure, unreliable markets, and limited extension services have constrained the sector's growth for many years (MOA and FAO 2018; Mrema et al. 2008; Rutta 2022). Ilula Ward, being one of the largest producers of tomatoes in Tanzania, Ikokoto, and Image villages were ideal study sites for examining and understanding the overall effects of climate variability on the postharvest management of tomatoes.

### 2.2 Data Collection and Analysis

Purposive and snowball sampling techniques were used to select the study sample (farmers) that represent the experiences of a large population. Criteria such as farmers' age, years in tomato farming, and gender were considered when selecting farmers to be interviewed and engaged in the study. Leaders from farmers' groups and local government extension officers were also consulted to ensure that study participants reflected the living experiences of small‐scale tomato farmers in the Ilula ward. Based on these criteria, 52 tomato farmers from both villages were selected for interviews. Semi‐structured in‐person interviews and Focus Group Discussions (FGDs) were the primary data collection methods. A semi‐structured questionnaire was used for individual face‐to‐face interviews with farmers (see Data S1—Interview and Focus Group Discussion [FGD] Guides). The semi‐structured interviews involved 40 farmers, 20 tomato farmers from each village. The questionnaire covered several issues focusing on farmers' postharvest management experience, including pre‐ and postharvest challenges related to the storage and transportation of tomatoes and the extent and factors leading to postharvest tomato losses. In‐depth discussions with selected farmers regarding climate change risks, threats, and their implications for handling, storage, and transportation of harvested tomatoes were conducted using FGDs. Two FGDs (see Data S1—Interview and Focus Group Discussion [FGD] Guides) were conducted with 12 tomato farmers (six in Image village and six in Ikokoto village). The interviews and FGDs were audio‐recorded to allow better analysis and postinterview transcription. Before the interviews and FGDs, participants were given a consent form to fill in, allowing the researcher to record the interviews. The interviews lasted about 90 min, while FGDs were conducted for almost 2 h.

A thematic data analysis method was used to generate findings for this study. In the thematic analytical approach, the researcher's goal is to establish themes and patterns reported by the study population (Braun and Clarke 2006). In doing so, the researcher establishes different meanings based on common patterns based on experiences and stories shared by the study participants (Braun and Clarke 2006; Percy et al. 2015). To establish these patterns, all the recorded interview transcripts were compiled and listened to multiple times to capture participants' stories and identify common patterns. The transcripts were then uploaded to the QSR NVIVO software, coded, and analyzed to establish common themes and subthemes in order of their significance and frequencies. Participants' stories and voices are also reflected using direct quotes and statements shared during interviews and FGDs to enhance research findings.

## 3 Results

### 3.1 Demographic Profile of Tomato Farmers

Though in Tanzania, vegetable crop farming has traditionally been done by female farmers, in both villages, tomato production was primarily dominated by male producers. Over three‐quarters (85%) of interviewed tomato farmers were male, and only a few (15%) were female (Table 1). The majority of female farmers were observed to be more involved in picking, sorting, and packing freshly harvested tomatoes before transporting them to local markets and other countries. The low participation of females in tomato production was attributed to increased production costs that have become unaffordable for most female farmers to engage in tomato farming. Other female farmers indicated that the constant need to buy pesticides to control pests and the labor‐intensive nature of growing and harvesting tomatoes make it difficult for them to engage in tomato production with limited financial resources. Additionally, farmers revealed that pest control costs accounted for most of the money they spent on producing tomatoes, constraining many local farmers, especially women, from growing tomatoes as a livelihood.

**TABLE 1:** Demographic characteristics of interviewed farmers (*n* = 40).

Category	Farmers interviewed
Age	
20–39	55%
40–59	42.5%
60+	2.5%
Gender	
Male	85%
Female	15%
Education level	
No formal education	15%
Primary education	67.5%
Secondary education	17.5%
Other	0.0%
Years involved in tomato farming	
1–3 years	20%
3–7 years	7.5%
> 7 years	72.5%
Land size (ownership)	
0–2 acres	67.5%
3–4 acres	22.5%
More than 4 acres	10.0%
Received training on postharvest management	
Yes	10%
No	90%

*Source:* Fieldwork (2021).

During the FDGs, it was revealed that with increasing tomato production costs driven by higher prices on fertilizers, pests and other farm inputs, many female farmers who once dominated tomato farming in the study villages were pushed out because they could not afford quality seeds, pesticides and had limited access to credit and hence abandon growing tomatoes and shift their focus in other income generating activities that were less costly. It was also noted that, as men had more control of land and other assets such as motorbikes they could use as collateral to access credit and loans from local microfinance institutions, the situation was very different for women from both villages whose access and land ownership has been restricted due to traditional customary rules on land ownership in Tanzania that tend to favor men than women. The financial barriers facing women in Image and Ikokoto villages are not unique to women in Tanzania but a common trend observed in many parts of Africa. According to the Food and Agriculture Organization (FAO), less than 15% of women in Sub‐Saharan Africa (SSA) have access to input supply and reliable credit, limiting their ability to advance and support their farming activities (FAO 2019). As such, male dominance among tomato farmers in this study underscores the critical need to improve access to capital for female farmers, especially those in rural areas where financial resources are limited.

More younger farmers were observed to engage in tomato farming, indicating increased youth engagement in agriculture as an income source. More than half (55%) of interviewed farmers were between the ages 20–39, and 43% were those between 40 and 59 years (Table 1), while those above 60 years were a small group (2.5%). Despite the age difference, most farmers were experienced tomato farmers with over 7 years of experience growing tomatoes as a livelihood. Significant involvement of younger farmers in tomato production could imply that tomato is an attractive crop for youth who may find tomato farming to be a lucrative agribusiness opportunity. Other factors accounting for increased youth engagement could be labor intensity and increased production costs in tomato farming, which older farmers cannot cope with and afford. With young people under 30 accounting for nearly 50% of Tanzania's population, increased youth engagement in tomato farming needs to be promoted, which could also be a better employment opportunity for rural youths in a sector traditionally dominated by adults and older people. However, further research is needed to establish driving factors and how to sustain rural youth involvement in farming over the long term.

### 3.2 Production of Tomato

In both villages, tomato was a major vegetable cash crop, followed by green peppers, cabbage, and white eggplant. Production is done on a smaller scale, with most farmers growing on less than 3 acres of farmland. Tomatoes are produced twice a year; December–March is a high production season, while during July–October, production slows down. Nearly all farmers practice irrigation farming with their farms close to rivers and other natural water sources and use diesel‐powered water pumps to draw water from these sources. Yield per acre varies from farmer to farmer and is influenced by several factors. Farmers reported that a one‐acre plot of tomatoes could yield up to 120 kg of fresh tomatoes. Once harvested, tomatoes are packed in bamboo baskets (*Tengas*), with one basket weighing about 50 kg, and sold at 30,000–35,000 Tsh, equivalent to $15/$17 United States Dollars (USD).

In both interviews and FGDs, farmers reported declines in tomato yields compared to past production cycles. Many complained of high production costs, believed to be increasingly fuelled by unfriendly weather conditions and persistent pest attacks, forcing farmers to use pesticides constantly. Farmers revealed they spend more on pest control to limit outbreaks of other crop diseases that have somehow become unmanageable. Most interviewed farmers believed the outbreak of pests and crop diseases that never existed before could be driven by warmer weather observed over the last few years in their community.

There are seasons you expect to harvest 40 Tengas, and you end up picking 24 Tengas; about half of Tengas get spoiled even before harvest time. You go to the farm and find the leaves are dry or have changed color, and many pre‐mature tomatoes have holes in their skin because of pests. This happens even after you have done multiple sprays of pesticides. There should be the treatment of pests to control pre‐harvest losses before even bringing storage facilities because much of the losses of tomatoes we experience happen before harvesting time. (FGD Participant, Ikokoto Village).

While the study primarily focused on postharvest tomato losses, farmers in both villages reported noticing diseases such as Early Blight (Alternaria solani), which affect tomato maturity, something they had not experienced in the past. Many believed the outbreak of these diseases could be linked to a longer period of warmer weather, which may directly influence the growth or incubation of pathogens, leading to such crop diseases. This observation is consistent with previous studies (Hossain et al. 2013; Ngowi et al. 2007) that also found that crop losses due to pests like African bollworm, Tuta Absoluta, and Bacterial canker have become a serious problem for small‐scale vegetable farmers in Africa, with evidence from studies linking these outbreaks with climate variability. Given small‐scale farmers' vulnerability to climate risks during the pre‐ and postharvest stages, regular training on cost‐effective pest and disease control practices will be critical to boosting tomato yields in Tanzania.

### 3.3 Postharvest Management of Tomato

#### 3.3.1 Harvesting and Handling of Tomatoes

**FIGURE 2:**
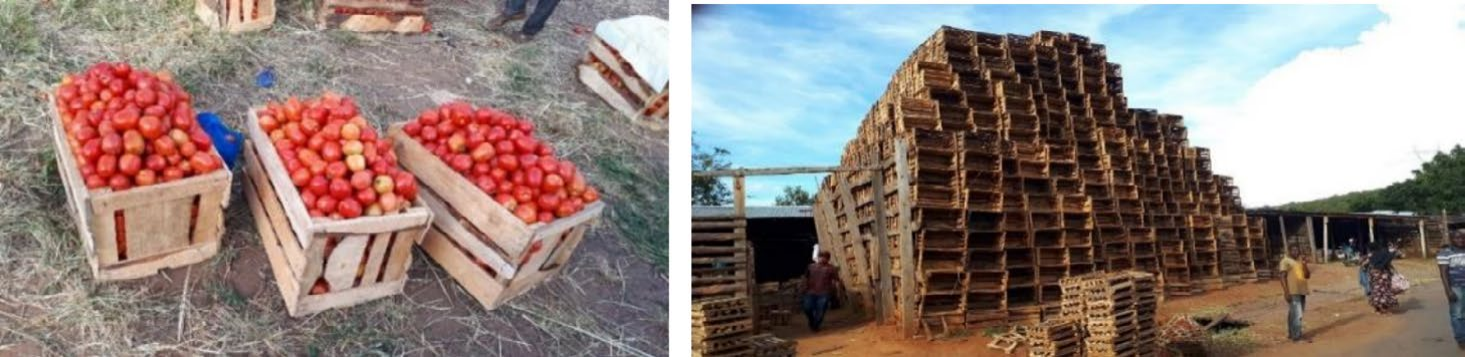
Wooden boxes (left) and stacked wooden boxes ready for sale in Ilula tomato market (right).

Tomatoes are harvested twice per week, with picking cycles changing depending on the maturity of tomatoes, weather conditions, and buyers' location. Tomatoes are often picked early in the morning, usually before 10 a.m., to avoid the heat that would spoil tomatoes before buyers collect them. Some buyers collect their tomatoes at the farm, while others place orders, and their tomatoes are transported to nearby marketplaces, such as the Ilula tomato market. Buyers outside Kilolo get their tomatoes picked a day earlier to save time and ensure they can be collected immediately from the farm when they arrive. Disposable Wooden boxes and Tengas (*Bamboo baskets*) were the most common packaging facilities used by all farmers to transport tomatoes to their destination markets near and far. The use of disposable wooden boxes and *Tengas* is attributed to their affordability. When visited the Ilula tomato market, the demand and broader use of disposable wooden boxes by tomato farmers and traders in Kilolo were very evident as packs of Disposable Wooden Boxes (Figure 2) were seen on public display stacked by local artisans waiting for farmers, traders and distant buyers to buy for packing tomatoes each sold for about USD $1. Though cheap and widely used in Tanzania, experts recommend farmers use more reliable packaging facilities that can protect fresh tomatoes from bruises and physical damage, mainly when transported to far distance (Kitinoja and Kader 2015; Mwagike and Mdoe 2015; Sibomana et al. 2016). However, in Tanzania, due to costs and access issues, plastic crates are expensive and hence rarely used by farmers, forcing many to rely on wooden boxes that somehow contribute to losses and spoilage of packed tomatoes when they reach their destination.

#### 3.3.2 Storage Losses Fueled by Weather Challenges

**FIGURE 3:**
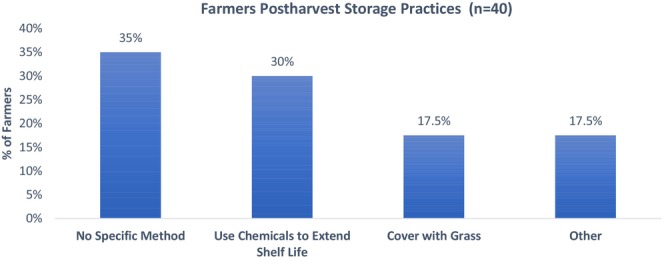
Storage practices employed by farmers.

Given the perishable nature of tomatoes, reliable storage is crucial to extending their shelf life before they are collected or transported to nearby markets (Kitinoja 2014; Yadav et al. 2014). Nearly all interviewed farmers reported having no dedicated storage facility for their fresh tomatoes after harvest. With no storage infrastructure, some farmers reported having no specific method and instead use traditional food preservation methods such water cooling to keep tomatoes fresh for a few hours or days while waiting for buyers to come (Figure 3). Others (30%) reported spraying some unknown chemicals to delay the ripening of the mature tomatoes while they seek potential buyers (Figure 3). Others reported covering harvested tomatoes outdoors with dry grass for a few days, hoping it does not rain. The lack of storage infrastructure left most harvested tomatoes more prone to spoilage as they were exposed to excessive heat for hours. As a result of poor storage conditions, farmers reported losing most of the harvested tomatoes when it rains and when potential buyers delay or fail to collect them.

During FGD discussions, farmers revealed that because rains have become irregular, losses linked to poor storage conditions have also been common. Some of their peers lose up to 80% of the tomato harvests when it rains and have nowhere to keep or sell mature tomatoes.

When we have frequent rains, tomato buyers from places like Dar es Salaam and other cities fail to come to collect tomatoes in Ilula. Unfortunately, during the rainy season, when we have a good harvest of tomatoes, almost everywhere you go in this village, every farmer will have tomatoes. And because we cannot predict the rains and have no place to store them until the rains slow down, we end up wasting our tomatoes in large quantities (FGD Participant, Ikokoto village).

Evidence shows that unfriendly weather conditions, such as erratic rains fueled by climate variability, will become normal and regular across Tanzania, putting the livelihoods of many farmers at risk (Abbass et al. 2014; Chegere 2018). For farmers engaged in perishable food products such as tomatoes, the availability of efficient on‐farm storage facilities will be critical to help them avoid postharvest tomato losses. Having a reliable storage infrastructure near farms and in tomato collection centers will enable farmers to keep their produce for a few days, especially when weather conditions significantly affect collection and the transportation of produce to markets. The availability of on‐farm storage facilities also helps farmers keep unsold tomatoes, reducing food wastage (Arah et al. 2015).

#### 3.3.3 Losses Linked to Transportation and Pick‐Up Delays

**FIGURE 4:**
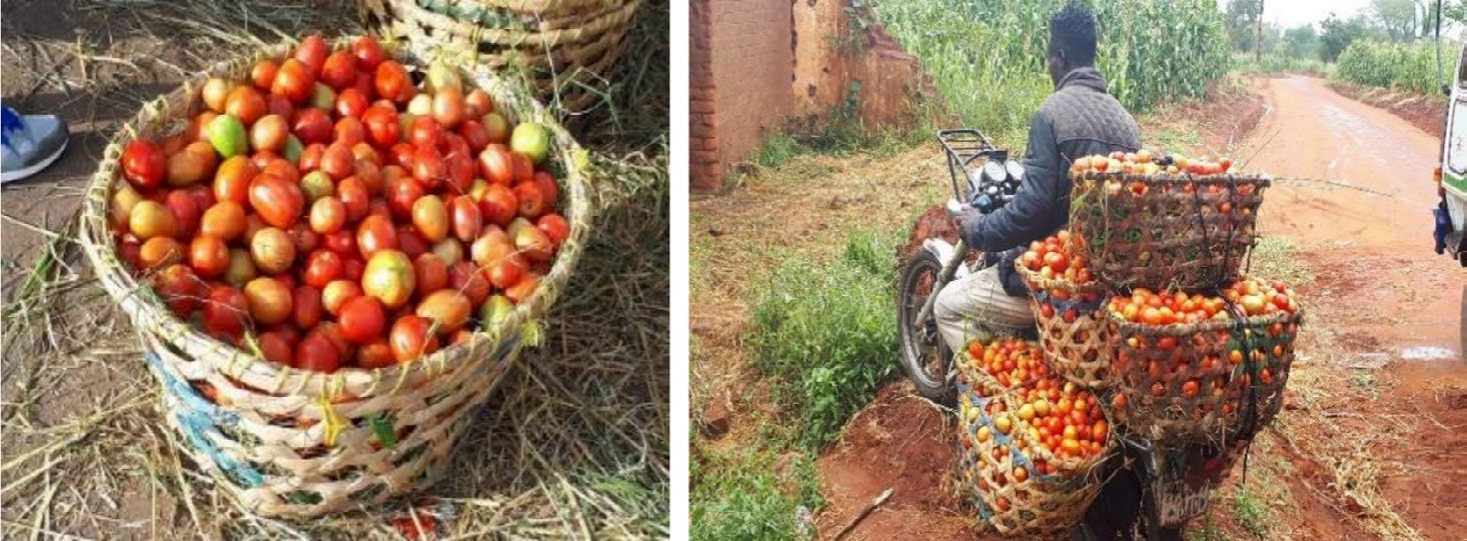
Fresh harvested tomatoes in Tengas transported to nearby markets.

Farmers in both villages reported that because of rains, delays of up to 2 days in collecting and transporting tomatoes to destination markets tend to happen more often, especially when dealing with buyers from Dar es Salaam, Tanga, and other distant markets. Though many of these buyers place their orders in advance, heavy rains and poor road conditions make it difficult for drivers to come and pick up tomatoes at the farm on time, with some drivers delaying for days. Since tomatoes for distant buyers are harvested a day or hours before transporters arrive, when such delays occur, farmers risk losing almost all harvested tomatoes unless an alternative buyer is available and willing to take uncollected tomatoes. This situation becomes more challenging because farmers have no storage facility to keep harvested tomatoes. Unsold tomatoes are normally kept under a shade for a few days; some get saved by transporting them with motorbikes to be sold at low prices to local buyers willing to buy them quickly before they spoil, while a significant volume of fresh, uncollected tomatoes ends up being thrown away (Figure 4).

#### 3.3.4 Losses Resulting From the Lack of Buyers

Farmers in Image village felt taken advantage of by local tomato traders, especially during the rainy season when the prices they offered were super low and did not match the production costs. Lower prices during the rainy season were reportedly driven by several factors, including scarcity of distant buyers from big cities within Tanzania and those from neighboring countries, particularly Kenya, unable to come to Kilolo due to weather‐related challenges, leading to an oversupply of tomatoes in Kilolo. Traders from Kenya were reported to collect tomatoes in large quantities compared to local buyers and offer better price margins. However, when rains become nonstop for longer periods of time, buyers outside Kilolo rarely come to collect tomatoes in Kilolo because of transportation challenges. This results in an oversupply of tomatoes and pushes prices down substantially from regular US $15 per Tenga to US $1 for a Tenga (35 kg) of fresh tomatoes. Even with such low prices, some farmers still find it difficult to sell their tomatoes, leading to waste.

It is always very sad to see almost all the tomatoes you have grown will have to be wasted because you have nowhere to keep them while you wait for a buyer (Farmer, Image Village).

While other factors also contribute to the drop in the price of fresh tomatoes, farmers in both villages believe the disruption of the tomato supply chain, especially transportation caused by weather challenges, is a major reason for the sharp decline in prices, especially when it rains. When this happens, unsold tomatoes tend to be wasted because there is no market.

#### 3.3.5 Gendered Impacts

For many years, women dominated the production of tomatoes and other vegetable crops in Tanzania partly because most vegetable crops have short production cycles and need less capital and land (FAOSTAT 2014; Fischer et al. 2020). However, this was not the case for both villages, as the study found that of the 40 tomato farmers interviewed, 85% were men (Table 1). Increasing production costs, especially regular purchase of pesticides to contain pests and crop diseases and paying laborers to help with postharvest operations like sorting, packing, and transporting tomatoes from farms to marketplaces, were mentioned to constrain many women in both villages to quit tomato farming. During FGD discussions, farmers described that one would need at least 2 million Tanzanian shillings (Tsh), equivalent to USD 1100 (as of June 2021), to kickstart tomato farming and get good yields. A significant portion of this money would be for pesticides and postharvest management costs that many women could not sustain because they lacked financial resources.

Last year, I had to sell my valuable items like TV and Motorbike because a large volume of tomatoes I produced could not be sold, and I ended up wasting them. As my finances dried, I couldn't afford to cover my children's expenses. Ultimately, I stopped growing tomatoes and went into green peppers because they don't need so much money as tomatoes. (Focus Group Participant, Ikokoto village).

Furthermore, increased weather challenges and tomato price fluctuations discouraged many women from growing tomatoes as a livelihood. In one interaction, female FGD participants revealed that after experiencing postharvest losses, they were left with the financial burden of paying back loans that they had taken after failing to sell their tomatoes. Eventually, these women decided to quit and switched to less costly vegetable crops like green peppers, cabbage, and eggplant, which are less prone to pests and diseases and require less capital.

## 4 Discussion

The results indicate that the effects of climate change are felt by farmers at almost every stage of tomato farming. Changes in weather conditions were noted to drive the growth and outbreak of pests and other crop diseases, forcing farmers to use pesticides, which became costly to many consistently. According to farmers, an outbreak of pests was fueled by warmer weather, creating favorable conditions for the rapid spread of known and unknown pests, leading to preharvest losses, which also exacerbated the tomato loss problem. In both villages, an increase in *Tuta Absoluta* pest attacks was believed to be linked to climate change. These findings correlate with several other previous studies that reveal that as local climate conditions continue to change in Tanzania, outbreaks of pests and crop diseases, such as “tomato lead miners,” will be common and may surge in many parts of Africa (Kariathi et al. 2016; Ngowi et al. 2007; Subedi et al. 2023). While pests driven by climate variability were a serious problem, interviewed farmers were noted to lack knowledge and training on effective pest management and hence fail to contain pests and any other crop diseases. Failure to contain pests, grown tomatoes become more vulnerable to preharvest losses in almost every season, leading to low yields and seriously affecting farmers' livelihoods (Kariathi et al. 2016; Ngowi et al. 2007).

In addition, due to their high perishability, it was noted that long periods of warmer weather present serious challenges in handling and postharvest management of tomatoes. Therefore, building tomato farmers' adaptive capacity to change in local climate conditions is crucial. Immediate interventions are thus needed to contain pest invasions, especially for tomatoes and other vegetable crops that have received limited attention. This must go hand in hand with regular extension support and training to farmers on the proper use of pesticides and enforcement of strict regulations to limit the adverse health effects of pesticides on consumers (Kariathi et al. 2016; Subedi et al. 2023). The financial burden of controlling pests was also noted to significantly impact female farmers, who were found to be disproportionately affected and further marginalized by the effects of climate change. Constrained by a lack of financial resources, most female farmers reported working as laborers on other people's farms while others engaged in petty trading for survival. This means that for female farmers, failure to adapt to changing climate conditions aggravated gender inequalities that already exist in farming across the continent (Bryan et al. 2024; Okesanya et al. 2024).

Furthermore, the study reveals that climate‐induced weather challenges, such as irregular rainfall and warmer temperatures, cause postharvest storage losses among tomato farmers. Without proper storage, harvested tomatoes are kept in shaded areas as farmers wait for buyers. This increases the risk of spoilage. In both villages, postharvest tomato losses are much higher during the rainy season. Farmers reported losing up to 40% during the rainy season. In contrast, losses are less than 20% in the dry season, when village roads are in good condition. This allows buyers from distant markets to collect most tomatoes soon after harvest. During the rainy season, the risk of losing most of the harvest increases because potential buyers do not show up, a problem mentioned by most farmers. If uncontrolled, these losses, mainly due to transportation delays, have a devastating impact on the livelihoods of small‐scale tomato farmers who rely on farming for income.

To avoid these storage losses, access to reliable cold storage infrastructure near tomato farms and collection centers is critical and could reduce unnecessary tomato waste, especially during unfriendly weather conditions (Rutta 2022). Since many of these farmers are in off‐grid areas, one practical and cost‐effective storage solution is a solar‐powered cold storage facility. Recently, solar‐powered cold storage facilities have been recognized as a viable solution to preventing storage losses facing African farmers on perishable produce value chains (Rutta 2022; Teutsch and Kitinoja 2019). In Nigeria and Kenya, local companies such as Cold Hubs and SokoFresh have paved the way for the introduction of solar‐powered cold storage infrastructure in the continent. To date, ColdHubs has deployed over 50 cold rooms benefiting thousands of farmers in Nigeria, proving the significance of cold storage infrastructure in preventing storage losses on perishables (Takeshima et al. 2022).

As climate‐induced weather challenges are predicted to become worse, postharvest spoilage on perishable food crops will likely increase across Africa (Chegere 2018; Kasso and Bekele 2018). The emergence of off‐grid cold storage facilities in Nigeria and Kenya is of prime significance in places like Kilolo, where farmers are more vulnerable to climate variability. Ensuring wider uptake of such facilities among farmers will be necessary. This, however, can only be realized with a supportive regulatory environment and innovative policy incentives that will bring much‐needed capital and investment in cold storage facilities for small‐scale farmers (Kitinoja 2014; Rutta 2022).

## 5 Conclusion

Climate change is arguably the greatest threat to African small‐scale farmers' livelihoods. While much of the climate change interventions in Africa remain focused on increasing crop yields, tackling climate change impacts must also address the risks and threats posed postproduction (Berck et al. 2018; Stathers et al. 2013). Postharvest agricultural adaptation interventions, therefore, become crucial because food production across Africa will likely decline due to weather variability. Similarly, ongoing climate mitigation and adaptation efforts must also shift their focus on enhancing farmers' adaptive capacity to reduce and prevent crop losses after harvest. For perishable crops such as tomatoes, this could come in different forms, including enabling farmers' access to low‐cost cold storage facilities, connecting farmers with reliable markets, and training farmers to forecast their production and harvest to reduce storage losses and losses resulting from lack of buyers (Teutsch and Kitinoja 2019; Kitinoja 2014; Stathers et al. 2020). Improving cold storage infrastructure should also be complemented with value‐added postharvest services, such as establishing small‐scale tomato processing machines enabling rural farmers to turn unsold tomatoes into tomato paste, juice, ketchup, and other products on demand by many urban consumers.

## Funding

The author has nothing to report.

## Ethics Statement

Research Ethics for this study was obtained at Tanzania's Commission of Science and Technology (COSTECH) with ethics approval number 2020‐351‐NA‐2020‐115‐29.

## Conflicts of Interest

The author declares no conflicts of interest.

## Supporting information


**Data S1:** pei370161‐sup‐0001‐Supinfo.docx.

## Data Availability

Data sharing not applicable to this article as no datasets were generated or analysed during the current study.
