# Supplementary material for: Climate‐Driven Food Loss: The Case of Postharvest Tomato Losses in Southern Tanzania
Source: Plant Environ Interact. 2026 Jun 10;7(3):e70161. doi: 10.1002/pei3.70161 (PMC13250695; doi:10.1002/pei3.70161)
Supplement: Supplementary file 1 — Data S1: pei370161‐sup‐0001‐Supinfo.docx. [file PEI3-7-e70161-s001.docx]

**INTERVIEW GUIDE FOR SMALL-SCALE TOMATO FARMERS**

**Kilolo District, Iringa, Tanzania**

**Name of the Respondent: ……………………………………… Village: ………………………………………**

**SECTION A: FARMER DEMOGRAPHIC INFORMATION**

A1. Age of Respondent. **1**. 20-39 years **2**. 40-59 years  **3**. 60+ years

A2. Gender **1**. Female **2.** Male

A3. Respondent’s Level of Education. **1.** No Formal Education [ ] **2.** Primary Education [ ] **3.** Secondary Education [ ]

**4.** Other [ ] ( Specify)

A4. How long have you been involved in Tomato Farming**? 1**. 1-3 years [ ] **2**. 3-7 years [ ] **3.** More than 7 years [ ]

A5**.** Besides Tomatoes, are there other vegetable crops you grow? **1.** Yes [ ] **2.** No [ ], If YES, which ones…………

A5. Are you farming other food crops? **1.** Yes [ ] **2.** No [ ], If **YES**, can you describe other crops you cultivate?

A6. Besides farming, are you involved in non-farm activities? **1**. Yes [ ] **2**. No [ ], if **YES**, can you describe which non-farm activities you are involved in?

A7. Can you describe to me of the vegetable crops you mentioned above, what percent of that is for trading and for household consumption?

A8. Do you keep record of your farming activities? **1.** Yes [ ] **2.** No [ ], if YES, can you describe to me how you keep your records?

A9. How would you describe your tomato production and sales outputs over the years?

**1.** Stagnant [ ] **2**. Decreasing [ ] **3.** Increasing [ ] **4**. Fluctuating [ ]

A.10. What do you think accounted for that…………………………………………………………………………….?

**SECTION B: FARMER’S POST-HARVEST PRACTICES**

**Research Question 1:** To what extent solar-powered post-harvest technologies will be suitable for farmers in Tanzania?

B1. Can you describe to me how you harvest your tomatoes?...................................

B2. On average how many Kilograms or *Tengas* of tomatoes do you harvest per season?

B3. Do you have market arrangement with traders before harvesting? **1**. Yes [ ] **2**. No [ ]

B4. Who does harvest of your tomatoes? **1.** Myself **2.** Trading Partners **3.** Family Members **4.** Farmer Association **5.** Other

B5. At what time of the day do you usually harvest your tomatoes? **1.** Morning[ ] **2**. Afternoon [ ] **3.** Evening [ ] **4.** No specific time [ ]

B6. Do you have any special reason for harvesting at a specific time of the day? **1.** Yes [ ] **2.** No [ ], explain

B7. How do you store and prevent spoilage of your tomatoes? and did you acquire any training in doing that?

B8. Do you experience any losses or spoilage of your tomatoes after harvest? **1.** Yes [ ] **2.** No [ ]

B9. Are you using any facility or technology to store you tomatoes and prevent spoilage? **1.** Yes [ ] (Specify...) **2.** No [ ]

B10. If yes, where is it located? **1.** On Farm  **2.** In the House **3.** Public Space **4.** Other

B11. What do you think causes postharvest losses of your tomatoes?

1. Poor Market Access [ ]
2. Unreliable means of Transport of produce to the market [ ]
3. Lack of storage Technology/Facility [ ]
4. Other (Specify)……………………

B12. On average what percent of your harvest is lost or spoiled during storage and Why?

B13. What have you been doing to prevent losses after harvest?

B.14. What do you do with tomatoes that get spoiled?

B15. What time of the year do you experience significant losses and spoilage of your tomatoes and Why?

B15. What packaging material do you use for your tomatoes?

**1**. Tenga [ ]

**2**. Wooden Boxes [ ]

**3**. Plastic Crates [ ]

**4**. Other [ ] (Specify)………………………………………………………

B16. How has crop losses or spoilage affected your income? How do you cope with that?

**SECTION C: FARMER’S ACCESS TO POST-HARVEST SERVICES AND EXTENSION**

**Research Question 2**: What are opportunities, barriers, and potential mechanisms towards dissemination of solar powered post-harvest technologies?

C1. Can you describe to me your relationship with the government Extension Officers?

C2. Do you get any form of support to avoid or prevent postharvest losses? **1**. Yes [ ] **2.** No [ ]

C3. Do farmers in this village receive training in postharvest management of tomatoes? **1**. Yes [ ] **2.** No [ ]

C4. If YES on **C2** & **C3**, who provides that support and training

**1**. Farmer Association [ ]

**2**. Government Extension Officers [ ]

**3.** NGOs [ ] (Which one……)

**4**. Other (specify)…………………………. [ ]

C5. Was the training and support you received beneficial to you and other farmers? Please describe your experience……………………………

C6. If NO, do you think you need such training? **1.** Yes [ ] **2**. No [ ]

C7. In your opinion, which post-harvest extension delivery approach would you prefer and Why?

**1.** Extension Visits [ ]

**2.** Farmer to Farmer Training [ ]

**3.** Demonstration [ ]

**4**. Other……… [ ]

C8. In your opinion, what is the most effective way to reach farmers with information about post-harvest management, and Why?

1. Extension visits [ ]
2. Radio [ ]
3. Telephone (WhatsApp/Sms) [ ]
4. Farmer Days [ ]
5. Other [ ]

C9. Have you received any facility or technology support to avoid postharvest losses for your tomatoes? **1.** Yes [ ] **2.** No [ ]

C10. Are you aware of any post-harvest facility or technology that has been introduced in this village? **1.** Yes [ ] **2**. No [ ], If yes which one and who introduced it?

C11. What facilities and technologies do you need most to prevent spoilage of your tomatoes and Why?

C12. Would you be willing to adopt/use innovative storage methods if they prove more effective than what you currently use?

**1.** Yes [ ] **2.** No [ ]

**SECTION D: FARMER’S OPINIONS ON POST-HARVEST TECHNOLOGIES**

**Research Question 3**: How can state and non-state actors enhance uptake/dissemination of solar-powered post-harvest technologies in Tanzania?

D1. Are you aware of solar powered cold chains technologies that prevent spoilage of fresh produce? **1.** Yes [ ] **2**. No [ ]

D2. What is your opinion regarding solar powered cold chain technologies?

D3. Have you considered to adopt or use solar powered cold chain technologies**? 1.** Yes [ ] **2.** No [ ]

D4. What do you think has been a barrier for you to adopt/use solar powered cold chain technologies?

1. Financial/Cost [ ]
2. Lack of Knowledge/Operational Skills [ ]
3. Uncertain of their Efficacy [ ]
4. Other[ ] ( Specify)

D5. Are there any risks that has/may affect your decision to use/adopt solar powered cold chain facilities? Please describe….

D6. Are you willing to adopt/use solar powered technologies if they are cost-effective, easy to use and manage?

1. Yes [ ] 2. No [ ]

D7. Can you describe to me the kind of support you will need to have access to solar powered cold chain facility?

D8. What do you think needs to be done to have farmers in this village access solar powered cold chain facilities?

*******************************************THE END*******************************************************

**INTERVIEW GUIDE FOR FOCUS GROUP DISCUSSION (FGD) WITH FARMERS**

**Group #: ……………… Group Composition: 1. Female……… 2: Male ………... Age Range:**

**Research Question 1: To what extent solar-powered post-harvest technologies will be suitable for farmers in Tanzania?**

1. How has post-harvest losses affected your incomes and local economy in this village? How do you cope with that?
2. Why do you think most farmers have no access to better post-harvest technologies? What can be done to resolve this situation?
3. Are you aware of any better post-harvest facility or technology that has been introduced in this village? If yes which one and who introduced it?
4. Which post-harvest technologies will be suitable for farmers in this village and Why?
5. Would you be willing to adopt/use innovative storage methods if they prove more effective than what you currently use? Why or Why not?

**Research Question 2: What are opportunities, barriers, and potential mechanisms towards dissemination of solar powered post-harvest technologies?**

1. Do you get any form of support to help you prevent postharvest losses? If Yes, from Who and how often?
2. Do you think regular post-harvest extension support could be a solution to post-harvest losses? How can this be done to benefit most farmers in this village?
3. How do you think potential post-harvest facilities or technologies should be introduced and disseminated to farmers in this village?
4. Are you aware of solar powered cold chain facilities that prevent spoilage of fresh produce? What is your view on these technologies?
5. Have you considered to adopt or use solar powered post-harvest technologies? If Yes/ No, WHY?
6. What do you think have been barriers for you to adopt/use solar powered COLD chain facilities?
7. How can such barriers be resolved?

**Research Question 3: How can state and non-state actors enhance uptake/dissemination of solar-powered post-harvest technologies in Tanzania?**

1. Can you describe to me the kind of support you will need to have access to solar powered cold chain facilities?
2. Are there any risks that has/may affect your decision to use/adopt solar powered post-harvest technologies? Please describe….
3. What are your opinions ways to ensure potential post-harvest facilities directly benefit farmers in this village?

*******************************************THE END*******************************************************

**INTERVIEW GUIDE FOR TOMATO TRADERS, KILOLO DISTRICT, IRINGA REGION**

**Name of the Respondent: ………………………………………… Gender: Male [ ] Female [ ]**

1. How long have you been trading tomatoes 1. Less than a year [ ] 2. 1 – 4 years [ ] 3. More than 5 years [ ]
2. Are you involved in any other income generating activity besides tomato trading 1. Yes [ ] (specify...) 2. No [ ]
3. How do you get your clients? Who are your primary clients (Wholesalers within Tanzania/ Wholesalers outside Tanzania or International Wholesalers?
4. Do you cultivate your own tomatoes? 1. Yes [ ] (specify...) 2. No [ ]
5. What is your relationship with tomato farmers in this village? How do you work with them?
6. Have you ever experienced a great loss of your tomatoes because of spoilage? 1. Yes [ ] 2. No [ ]
7. Has this situation been recurrent? Yes [ ] No [ ]
8. Which of the following accounted for the losses of your tomatoes?

1. Unreliable Market [ ]

2. Lack of Storage Facilities [ ]

3. Poor Transport [ ]

4. Pest Attacks [ ]

5. Other [ ] (specify)……….

1. How would you assess the rate of tomato spoilage in your area over the years?

1. Very high [ ]

2. High [ ]

3. Low [ ]

4. Very low [ ]

5. Fluctuating [ ]

1. Are you often able to prolong the shelf life of your goods? 1. Yes [ ] (specify)………. 2. No [ ]
2. Do you own or have access to any storage facility? 1.Yes [ ] 2. No [ ]
3. Are you aware of any post-harvest technologies or facilities to prevent losses on fresh produce? Yes [ ] No [ ]
4. Are you aware of solar powered COLD chain facilities? 1. Yes [ ] 2. No [ ]
5. What is your opinion regarding solar powered cold chain facilities?
6. Have you considered to adopt or use solar powered post-harvest technologies? 1. Yes [ ] 2. No [ ]
7. What do you think has been a barrier for you to adopt of solar powered COLD chain facilities?
8. Why do you think solar powered technologies or facilities have not reached tomato farmers in this village?
9. What would be the best way to introduce and disseminate needed solar post-harvest technologies in this community?
10. In your views, what is the role of the government towards the introduction potential solar powered COLD chain facilities? And how can this be effectively done?
11. In what ways non-government players could help in the dissemination of potential solar powered COLD chain facilities? And how can this be effectively done to benefit farmers and traders?

*****************************************THE END*****************************************************

**INTERVIEW GUIDE FOR KEY INFORMANTS**

**Name of the Respondent: …………………………. Institution…………………………… Position: ……………………**

1. Can you please describe how your institution is working with small-scale horticultural farmers in Tanzania?
2. In what ways do you engage horticultural farmers in post-harvest management?
3. Do you provide any technical support to farmers in form of facilities or technologies to prevent crop losses after harvest? If yes, describe?
4. Are you aware of any post-harvest technologies in use by horticultural farmers to prevent PHL? How effective are they?
5. From your experience, which post-harvest technologies are needed and will be appropriate for horticultural farmers in Tanzania and Why?
6. Why do you think such technologies have not reached the majority farmers in Tanzania?
7. Are you aware of solar powered post-harvest facilities/technologies that prevent spoilage of fresh produce? What is your view on solar powered technologies?
8. How can such technologies be introduced and disseminated to rural farmers? What would be the government role in this process?
9. In many cases affordability of technologies has often been a barrier for adoption of agriculture technologies? How can this barrier be addressed?
10. In what ways non-government players could help improve access and dissemination of solar powered post-harvest facilities to small-scale farmers? And how can this be effectively done to benefit small-scale farmers?
11. What are your views on using market-based approach to introduce post-harvest facilities to small-scale farmers? How can be it be a win-win for everyone if implemented?
12. What are your opinions on policy strategies to ensure solar powered post-harvest technologies benefit rural small-scale farmers?

*******************************************THE END*******************************************************
